# Value of intralesional and perilesional radiomics for predicting the bioactivity of hepatic alveolar echinococcosis

**DOI:** 10.3389/fonc.2024.1389177

**Published:** 2024-06-27

**Authors:** Simiao Zhang, Juan Hou, Wenwen Xia, Zicheng Zhao, Min Xu, Shouxian Li, Chunhui Xu, Tieliang Zhang, Wenya Liu

**Affiliations:** ^1^ Imaging Center, The First Affiliated Hospital of Xinjiang Medical University, Urumqi, Xinjiang, China; ^2^ Imaging Center, The Friendship Hospital of Ili Kazakh Autonomous Prefecture, Yining, Xinjiang, China; ^3^ CT Scientific Collaboration Department, CT Business Unit, Canon Medical Systems (China) CO., LTD., Beijing, China

**Keywords:** computed tomography, hepatic alveolar echinococcosis, radiomics, perilesional, nomogram

## Abstract

**Objectives:**

To investigate the value of intralesional and perilesional radiomics based on computed tomography (CT) in predicting the bioactivity of hepatic alveolar echinococcosis (HAE).

**Materials and methods:**

In this retrospective study, 131 patients who underwent surgical resection and diagnosed HAE in pathology were included (bioactive, n=69; bioinactive, n=62). All patients were randomly assigned to the training cohort (n=78) and validation cohort (n=53) in a 6:4 ratio. The gross lesion volume (GLV), perilesional volume (PLV), and gross combined perilesional volume (GPLV) radiomics features were extracted on CT images of portal vein phase. Feature selection was performed by intra-class correlation coefficient (ICC), univariate analysis, and least absolute shrinkage and selection operator (LASSO). Radiomics models were established by support vector machine (SVM). The Radscore of the best radiomics model and clinical independent predictors were combined to establish a clinical radiomics nomogram. Receiver operating characteristic curve (ROC) and decision curves were used to evaluate the predictive performance of the nomogram model.

**Results:**

In the training cohort, the area under the ROC curve (AUC) of the GLV, PLV, and GPLV radiomic models was 0.774, 0.729, and 0.868, respectively. GPLV radiomic models performed best among the three models in training and validation cohort. Calcification type and fibrinogen were clinical independent predictors (p<0.05). The AUC of the nomogram-model-based clinical and GPLV radiomic signatures was 0.914 in the training cohort and 0.833 in the validation cohort. The decision curve analysis showed that the nomogram had greater benefits compared with the single radiomics model or clinical model.

**Conclusion:**

The nomogram model based on clinical and GPLV radiomic signatures shows the best performance in prediction of the bioactivity of HAE. Radiomics including perilesional tissue can significantly improve the prediction efficacy of HAE bioactivity.

## 1 Introduction

Alveolar echinococcosis is a zoonotic parasitic disease caused by the larva stage of *Echinococcus multilocularis*, which is popular in pastoral areas. The liver is the most commonly involved organ. The hepatic alveolar echinococcosis (HAE) presents as a slow-growing multiple cystic invasive mass, which is surrounded by macrophages, lymphocytes, and fibrocytes, and forms granuloma tissues resembling a malignant tumor; hence, it is called “worm cancer” (1). Currently, the preferred treatment of HAE is radical hepatectomy. However, HAE shows no obvious symptoms in the early stage; most patients are diagnosed late, as they already have large hepatic lesions with vascular or biliary structure involvement. Those patients have to receive a liver transplant or a long-term treatment with the drug. Therefore, it is important to identify the bioactivity of the lesion, which means the ability of the hydatid tapeworm to survive and to invade outward, as early as possible to determine the treatment strategy of the patients.

In the early 1990s, Wen et al. (2) proposed that there was an “infiltration zone” around HAE lesions, which formed granuloma tissues with no clear margins with the adjacent normal liver parenchyma, and determined the occurrence and expansion of HAE. Studies found that microvascular infiltration exists in the area of HAE, which is likely involved in the trafficking of immune cells and can be used to assess its bioactivity (3). However, it is challenging to quantify the microvessel density (MVD) preoperatively. Although researchers found that the proliferation and bioactivity of HAE lesions can be evaluated by CT perfusion, dual-energy CT imaging, diffusion-weighted magnetic resonance imaging (MRI), and positron emission tomography/computed tomography (PET/CT), these functional imaging techniques were difficult to popularize, as HAE was prevalent in less developed pastoral areas (4–6).

Radiomics incorporates a series of computational technologies. It can extract large amounts of high-dimensional quantitative features from multimodality medical images, then the mining of correlations between these features, and the diagnosis or prognosis of disease, which provides quantitative and objective support for disease detection and treatment (7, 8). A systematic review and meta−analysis found that radiomics models showed promising prediction performance for predicting microvascular invasion (MVI) in hepatocellular carcinoma (HCC) (9). Ren et al. (10) had developed and validated a radiomics model as an adjunct tool to predict the HAE bioactivity by combining T2-weighted imaging (T_2_WI), which achieved results nearly equal to the PET/CT findings. However, there were no studies on the perilesional radiomics of HAE. The present study aimed to explore the perilesional radiomics based on CT in predicting the bioactivity of HAE.

## 2 Materials and methods

This retrospective study was approved by the institutional Ethics Committee of the First Affiliated Hospital of Xinjiang Medical University (No. K202312–38), and patient informed contents were waived.

### 2.1 Study population

The pathological database of the First Affiliated Hospital of Xinjiang Medical University between January 2015 and December 2021 was searched. The inclusion criteria were as follows: (1) HAE was confirmed by pathology; (2) the bioactivity of HAE lesions was determined; (3) contrast-enhanced abdominal CT was performed within 2 weeks before surgery; and (4) CT images was complete and met the diagnostic requirements. The exclusion criteria were as follows: (1) clinicopathological record was incomplete; (2) the liver lobes were abnormally developed; (3) there were other malignant tumors; (4) interventional therapy before surgery was performed; and (5) the lesion edges were not easily segmented.

Finally, a total of 131 patients were eligible. All patients were randomly divided into training cohort and validation cohort at the ratio of 6:4 (training cohort, n=78; validation cohort, n=53), respectively.

### 2.2 Pathological diagnosis of bioactivity

According to previous studies, perilesional MVD reflects the bioactivity of the HAE (3). Two physicians with 10 years of pathological diagnosis who were blinded to the information of patients interpreted the MVD of all patients independently. First of all, each section with CD34-stained tissue was processed at low magnification (×40) to spot three hotspots representing the local areas of the highest microvessel density. Second, MVD was counted with high magnification (×200) for clear imaging and better counting. The mean count of the large and small microvessels in each of the three hotspot areas was the final MVD. The MVD > 15 was considered bioactive for HAE.

### 2.3 Clinical model construction

Clinical features included age, sex, smoking history, calcification type, and some relevant laboratory tests, such as white blood cells (WBC), neutrophils count (NE), lymphocyte count (LY), fibrinogen (Fib), platelet count (PLT), creatinine (Crea), total bilirubin (TBIL), aspartate aminotransferase (ASL), alanine aminotransferase (ALT), and prothrombin time (PT). The independent predictors from traditional features were identified by univariate and multivariate logistic regression for the construction of clinical model.

### 2.4 Image acquisition

All enrolled patients have taken CT examinations with the following two scanners: Aquilion ONE Genesis Edition (Canon Medical Systems, Japan) and GE Discovery HD750 (GE Medical Systems, Milwaukee, WI, USA). Patients were injected with iodinated contrast material (350 mg I/mL, 1.5 mL/kg) through the median cubital vein using a double-head power injector (injection rate, 3.0 mL/s), followed by a 30-mL saline flush at the same injection rate. The artery phase was started immediately when the CT value of the region of interest (ROI) reached the threshold of 180 HU. The portal venous phase and delayed phase were obtained at 50–70 s and 90–120 s, respectively, after injection of contrast.

### 2.5 Lesion segmentation and features extraction

**Figure 1:**
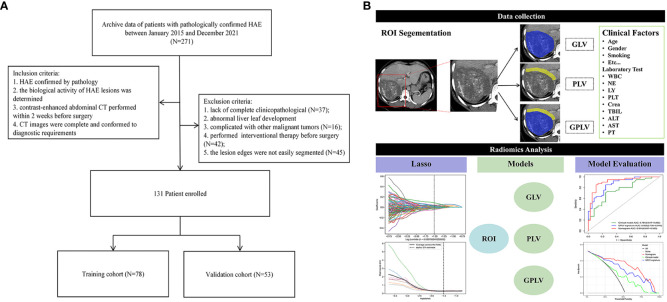
**(A)** Flow chart of patient enrollment; **(B)** the radiomics workflow.

**Figure 1B** shows the workflow of this study. Two radiologists with 5 years and 10 years of experience, who were blinded to the patients’ clinical information, depicted the volume of interest (VOI) of lesion in portal venous phase. If there was more than one lesion in the liver, the largest one was selected. Before segmentation, the gray value of all images was standardized. The extracted features were standardized using z-score normalization, which adhere to each feature with a mean of 0 and a standard deviation of 1. The edge of the lesion was marked by manual operation, and the perilesional was defined as the liver parenchyma within a distance of 10 mm outside the lesion edge, which was automatically segmented by 3D Slicer (version 4.13.0). The detailed steps were as follows: imported the axial images with a layer thickness of 5 mm into the software, marked the lesion edges layer by layer for the VOI of GLV with the CT value ranged from 200 HU to 500 HU, copied the GLV as a new segmentation, and grew by specified margin size of 10 mm. The final step was to erase the blood vessels and bile ducts and anything beyond the liver to get the VOI of PLV.

The radiomics features were then extracted from each VOI using open-source Pyradiomics (version 3.0.1, https://pyradiomics.readthedocs.io/en/latest/index.html) toolbox and processed using Scikit-learn (version 1.0.2, https://scikit-learn.org/stable/index.html) package.

### 2.6 Feature selection and radiomics model construction

First, CT images of 30 patients were selected randomly to evaluate the inter-reader consistency of radiomics features labeled from the two radiologists. The ICC >0.9 was considered reproducible features, and the remaining features were excluded in the further analysis.

Next, the univariate analysis (one-way ANOVA) was used to assessed statistical difference between features and bioactivity, and only features with a p-value <0.05 was retained. The least absolute shrinkage and selection operator (LASSO) was performed to further select the most significant features. A 10-fold cross-validation was applied to determine the optimal lambda value, minimizing the prediction error.

Five conventional machine learning algorithms was used to illustrate the stability and robustness of the performance of the models, including support vector machine (SVM), logistic regression (LR), random forest (RF), decision tree (DT), and k nearest neighbors (KNN). The best classifier was chosen according to the mean AUC results obtained from a 10-fold cross-validation with 100 iterations.

### 2.7 Clinical radiomics nomogram construction

The statistical difference of patient baseline characteristics and HAE-related clinical factors according to univariate and multivariate analyses were performed to identify the independent predictors. The best radiomics model and the predictors were then used to construct the nomogram based on the logistic regression. In addition, the nomogram facilitates prediction by summing the points corresponding to all predictors, thereby determining the risk of bioactivity. It offers a direct and visual representation of the assessment for bioactivity of HAE. The predictive accuracy of the nomogram was assessed using the Hosmer–Lemeshow test. The decision curve analysis (DCA) was performed to assess the clinical utility of the nomogram.

### 2.8 Statistical analysis

Statistical analyses were performed using R software (version 4.2.2; http://www.R-project.org). General information were expressed as the mean ± standard deviations (SD) or frequency. The chi-square test was used for categorical variables. The ICC value of concordance was used to measure the degree of agreement between the two experienced radiologists. The independent predictors of the bioactivity of HAE were determined by univariable and multivariable logistic regression analysis. Bilateral p-values < 0.05 were considered statistically significant. The ROC curve was drawn, and the AUC was calculated to evaluate the diagnostic performance of each model. DeLong test was used to assess differences in AUC between models.

## 3 Results

### 3.1 Clinical features

The flowchart of patients enrolled is shown in **Figure 1A**. A total of 131 patients were included (bioactive, n=69; bioinactive, n=62). The training and validation cohort included 78 and 53 patients, respectively. The clinical features of the two cohorts are shown in **Table 1**. There were no statistically significant differences in age, sex, smoking history, WBC, NE, LY, PLT, Crea, TBIL, ASL, ALT, and PT between the training cohort and validation cohort (all p > 0.05). Both univariate and multivariate logistic regressions showed statistical difference in calcification type and fibrinogen (p<0.05), which were selected to established the clinical model (**Table 2**).

**Table 1:** Baseline patient characteristics in training and validation cohort.

Characteristics	Training cohort			Validation cohort		
	BA-active (n = 41)	BA-inactive (n = 37)	*p*-value	BA-active (n = 28)	BA-inactive (n = 25)	*p*-value
Age			0.862			0.823
≤50 years	36	32		22	19	
>50 years	5	5		6	6	
Sex			0.764			0.232
Male	18	15		10	13	
Female	23	22		18	12	
Smoking			0.274			0.487
No	16	19		13	14	
Yes	25	18		15	11	
Calcification type			<0.001			<0.001
I	2	13		1	12	
II	23	20		15	11	
III	16	4		12	2	
WBC			0.848			0.883
≤9.5×10^9^/L	36	33		25	22	
>9.5×10^9^/L	5	4		3	3	
NE			0.561			0.122
≤6.3×10^9^/L	39	34		27	21	
>6.3×10^9^/L	2	3		1	4	
LY			0.368			0.486
≤3.2×10^9^/L	35	34		27	23	
>3.2×10^9^/L	6	3		1	2	
Fib			<0.001			<0.001
≤4 g/L	39	14		23	3	
>4 g/L	2	23		5	22	
PLT			0.731			0.471
≤350 g/L	38	35		24	23	
>350 g/L	3	2		4	2	
Crea			0.116			0.340
≤110 µmol/L	36	36		27	25	
>110 µmol/L	5	1		1	0	
TBIL			0.070			0.205
≤22 µmol/L	27	31		18	20	
>22 µmol/L	14	6		10	5	
AST			0.220			0.275
≤59 U/L	27	29		20	21	
>59 U/L	14	8		8	4	
ALT			0.092			0.552
≤72 U/L	29	32		23	22	
>72 U/L	12	5		5	3	
PT			0.560			0.805
≤38 s	27	22		17	16	
>38 s	14	15		11	9	

BA, bioactivity; WBC, white blood cells; NE, neutrophils count; LY, lymphocyte count; Fib, fibrinogen; PLT, platelet count; Crea, creatinine; TBIL, total bilirubin; ASL, aspartate aminotransferase; ALT, alanine aminotransferase; PT, prothrombin time.

**Table 2:** Logistic regression analysis of clinical factors of patients in the training cohort.

Factors	Univariate analysis		Multivariate analysis	
	OR (95% CI)	*p*-value	OR (95% CI)	*p-*value
Age	1.022 (0.981–1.065)	0.298		
Sex	0.871 (0.354–2.144)	0.764		
Calcification level	4.818 (1.996–11.632)	<0.001	4.207 (1.376–12.861)	0.012
Smoking	1.649 (0.671–4.055)	0.276		
WBC	1.022 (0.884–1.182)	0.764		
NE	0.957 (0.741–1.237)	0.738		
LY	1.169 (0.899–1.521)	0.244		
Fib	0.153 (0.066–0.352)	<0.001	0.133 (0.049–0.359)	<0.001
PLT	0.999 (0.994–1.004)	0.624		
Crea	1.017 (0.990–1.045)	0.230		
TBIL	1.027 (0.998–1.057)	0.064		
AST	1.015 (0.999–1.032)	0.065		
ALT	1.006 (0.998–1.015)	0.133		
PT	1.101 (0.942–1.285)	0.226		

### 3.2 Radiomics analysis

**Figure 2:**
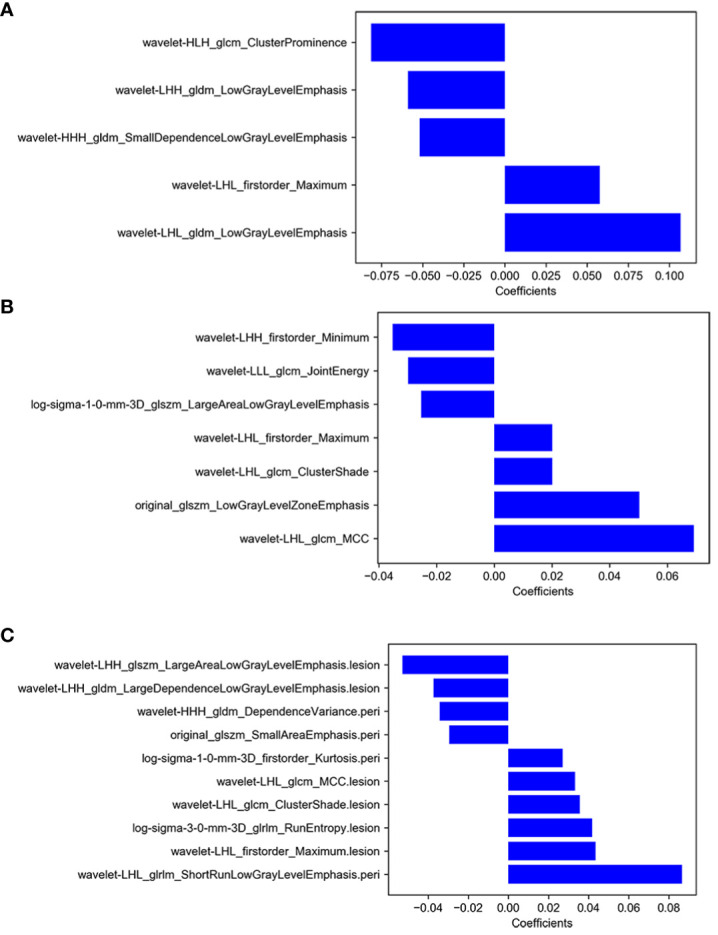
**(A)** The selected features of GLV model, **(B)** the selected features of PLV model, **(C)** the selected features of GPLV model.

A total of 1,037 radiomics features were extracted from the images for each patients, including 14 shape features, 18 first-order features, and 75 high-order features, containing 24 gray-level co-occurrence matrix (GLCM) features, 16 gray-level run length matrix (GLRLM) features, 16 gray-level size zone matrix (GLSZM) features, 14 gray-level dependence matrix (GLDM) features, and five neighboring gray tone difference matrix (NGTDM) features. These features were initially extracted from the original images, yielding a total of 107 features. Additionally, excluding shape features, eight wavelet transform filters and two Laplacian of Gaussian filters were applied to the first- and high-order features to generate (18 + 75) × 10 = 930 filtered features. A total of 974 features had good reliability with ICC > 0.90. After ANOVA, there were 162, 52, and 340 features selected with GLV, PLV, and GPLV, respectively. Finally, a total of 7, 5, and 10 optimal radiomics features were selected by applying LASSO method, respectively (**Figure 2**). Detailed information regarding the feature classes and names is shown in **Table 3**.

**Table 3:** ICC of the selected features in the three models.

Model	Feature			ICC
	Image type	Feature class	Feature name	
GLV	wavelet-LHL	glcm	MCC	0.906
	wavelet-LHL	glcm	ClusterShade	0.958
	wavelet-LHL	firstorder	Maximum	0.915
	wavelet-LHL	firstorder	Minimum	0.930
	Wavelet-LLL	glcm	JointEnergy	0.913
	original	glszm	LowGrayLevelZoneEmphasis	0.918
	log-sigma-1–0-mm-3D	glszm	LargeAreaLowGrayLevelEmphasis	0.954
PLV	wavelet-LHL	firstorder	Maximum	0.915
	wavelet-LHL	gldm	LowGrayLevelEmphasis	0.927
	wavelet-LHH	gldm	LowGrayLevelEmphasis	0.936
	wavelet-HHH	gldm	SmallDependenceLowGrayLevelEmphasis	0.935
	wavelet-HLH	glcm	ClusterProminence	0.943
GPLV	wavelet-LHL	glcm	MCC	0.906
	wavelet-LHL	glcm	ClusterShade	0.958
	wavelet-LHL	glrlm	ShortRunLowGrayLevelEmphasis	0.942
	wavelet-LHL	firstorder	Maximum	0.915
	wavelet-LHH	gldm	LargeDependenceLowGrayLevelEmphasis	0.942
	Wavelet-LHH	glszm	LargeAreaLowGrayLevelEmphasis	0.939
	wavelet-HHH	gldm	DependenceVariance	0.903
	original	glszm	SmallAreaEmphasis	0.908
	log-sigma-1–0-mm-3D	firstorder	Kurtosis	0.946
	log-sigma-3–0-mm-3D	glrlm	RunEntropy	0.908

The prediction performances of different machine learning models in training and validation cohort are shown in **Table 4**, which were averaged after 100 iterations. In general, all the machine learning models performed well, and the SVM showed the highest AUC both in the two cohorts (training cohort, 0.791; validation cohort, 0.776).

**Table 4:** Prediction performance of different machine learning models in training and validation cohort.

Classifiers	Training cohort				Validation cohort			
	AUC (95% CI)	Accuracy	Sensitivity	Specificity	AUC (95% CI)	Accuracy	Sensitivity	Specificity
SVM	0.791 (0.781–0.802)	82.60%	75.30%	90.30%	0.776 (0.766–0.786)	73.80%	71.30%	76.60%
KNN	0.765 (0.754–0.775)	77.20%	70.50%	84.20%	0.716 (0.706–0.726)	67.70%	65.10%	70.50%
RF	0.711 (0.699–0.723)	77.60%	69.00%	86.90%	0.685 (0.673–0.697)	68.20%	67.40%	69.20%
DT	0.729 (0.718–0.740)	75.10%	67.70%	83.20%	0.647 (0.634–0.661)	64.70%	61.60%	68.10%
LR	0.783 (0.773–0.794)	81.50%	74.70%	88.70%	0.761 (0.752–0.771)	72.60%	69.60%	72.50%

SVM, support vector machine; KNM, k-nearest neighbor); RF, random forest; DT, decision tree; LR, logistic regression; AUC, the area under the curve.

The AUC of GLV were 0.774 (95% CI, 0.669–0.878) and 0.736 (95% CI, 0.602–0.870) in training cohort and validation cohort, respectively. The AUCs of PLV were 0.729 (95% CI, 0.618–0.840) and 0.674 (95% CI, 0.528–0.820) in training cohort and validation cohort, respectively. The GPLV had the highest AUC among the three models: 0.868 (95% CI, 0.790–0.945) in the training cohort and 0.777 (95% CI, 0.652–0.903) in the validation cohort. As shown in **Table 5**, the sensitivity and specificity of GPLV were 0.854 and 0.757 in the training cohort and 0.736 and 0.750 in the validation cohort, respectively. Therefore, the GPLV model performs best in predicting the bioactivity of HAE; it can be inferred that the radiomics feature of PLV can be an important auxiliary tool for the diagnosis and treatment of HAE.

**Table 5:** Prediction performance of three models in training and validation cohorts.

Model	AUC (95% CI)	Accuracy	Sensitivity	Specificity	PPV	NPV	Youden index
GLV							
Training cohort	0.774 (0.669–0.878)	0.744	0.780	0.703	0.744	0.743	0.483
Validation cohort	0.736 (0.602–0.870)	0.698	0.734	0.690	0.715	0.729	0.424
PLV							
Training cohort	0.729 (0.618–0.840)	0.679	0.688	0.692	0.733	0.711	0.380
Validation cohort	0.674 (0.528–0.820)	0.660	0.621	0.680	0.706	0.639	0.301
GPLV							
Training cohort	0.868 (0.790–0.945)	0.808	0.854	0.757	0.795	0.824	0.611
Validation cohort	0.777 (0.652–0.903)	0.736	0.750	0.720	0.739	0.731	0.470

GLV, gross lesion volume; PLV, perilesional volume; GPLV, gross combined perilesional volume; PPV, positive predictive value; NPV, negative predictive value.

### 3.3 Clinical radiomics nomogram evaluation

**Figure 3:**
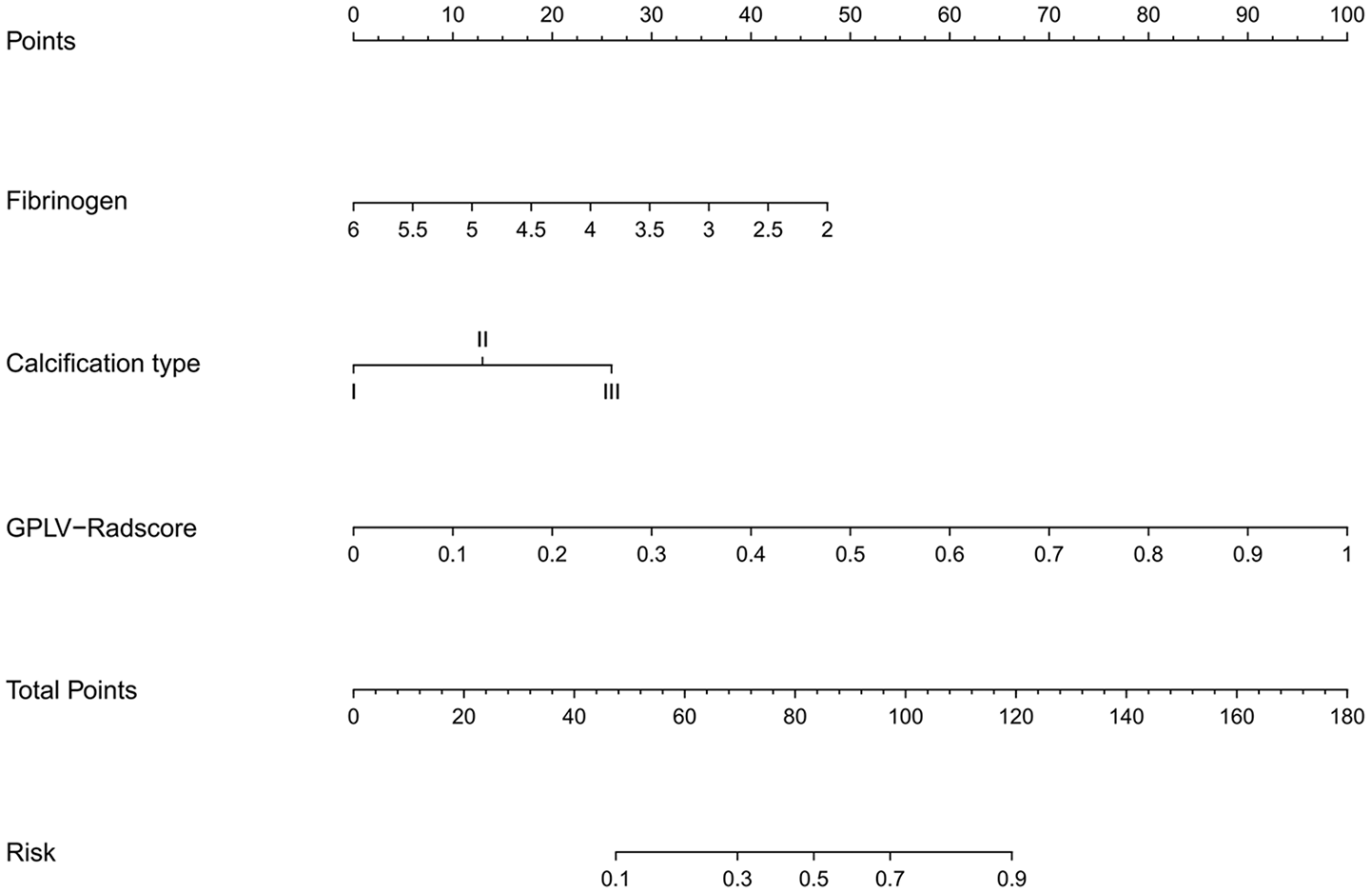
An individualized nomogram on Radscore and clinical features. The variables fibrinogen, calcification type, and Radscore have their own independent scale as shown in the figure, representing their respective value ranges. Each variable has a corresponding single item score at different values. The total score, summing each value of the variable, corresponds to the risk of bioactivity.

**Figure 4:**
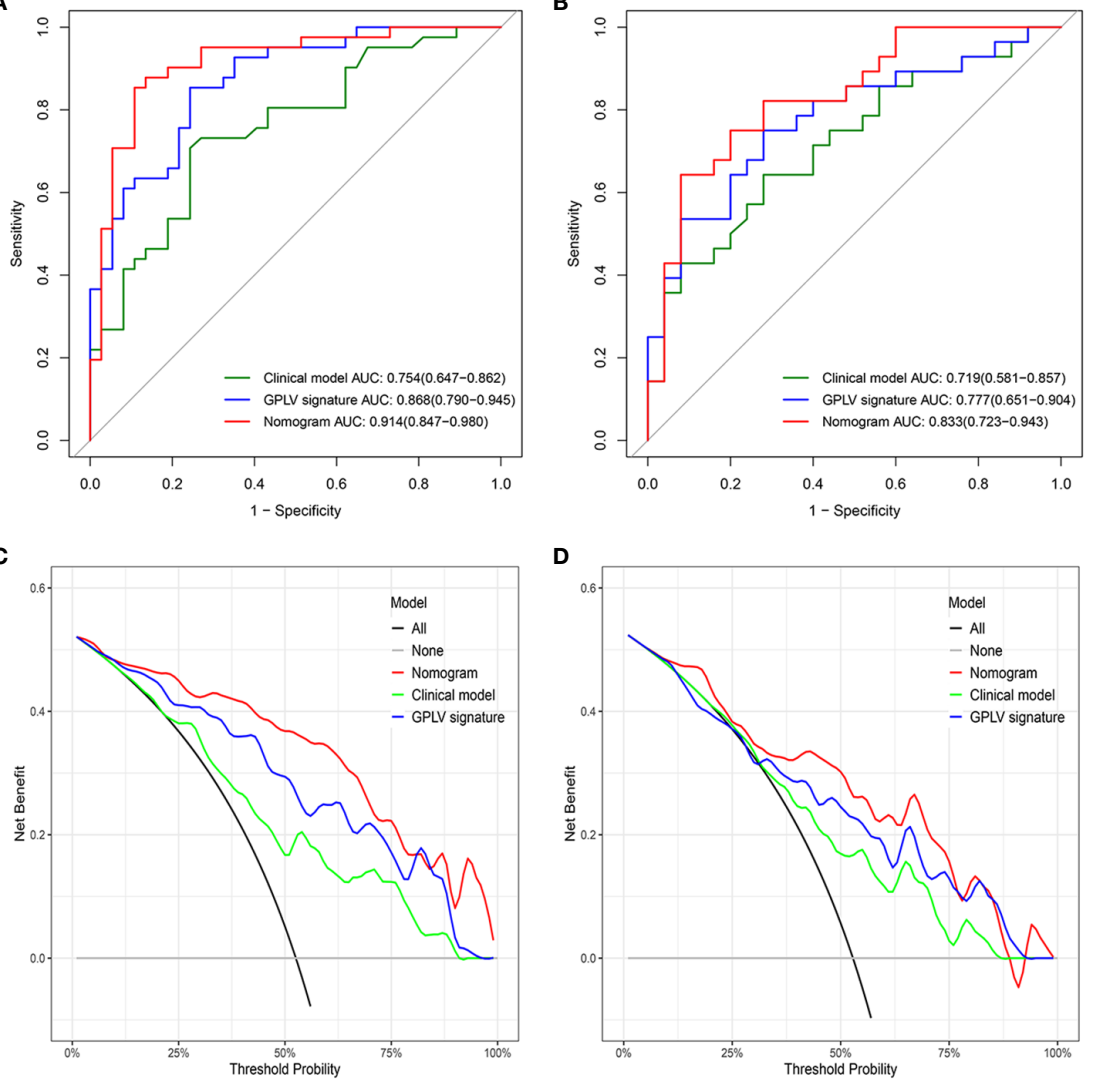
**(A)** The ROC curve for the three models in the training cohort, **(B)** the ROC curve for the three models in the validation cohort, **(C)** the DCA curve for the three models in the training cohort, **(D)** the DCA curve for the three models in the validation cohort.

The GPLV-Radscore, calcification type, and fibrinogen were incorporated into the nomogram (**Figure 3**). **Figures 4A, B** shows that clinical model, GPLV radiomics model, and nomogram had an AUC of 0.754, 0.868, and 0.914 in the training cohort, respectively. Similarly, the AUCs in the validation cohort were 0.719, 0.777, and 0.833 in the three models, respectively. Delong test showed that the AUC of the nomogram was significantly different from the clinical model and GPLV radiomics model (all p < 0.05). Notably, the DCA curves showed that the nomogram had greater benefits compared with the single radiomics model or clinical model (**Figures 4C, D**).

## 4 Discussion

This retrospective study explored the clinical value of perilesional radiomics based the portal venous phase of CT images in predicting bioactivity of HAE. The results showed that the radiomics model that integrates the lesion and surrounding tissue has the best predictive value. Then, we established a nomogram based on Radscore of the best radiomics model and independent predictors of clinical model; it had shown higher application value in predicting the bioactivity of HAE.

Radical surgical resection is the main treatment for HAE (11). Expert consensus suggests a 20-mm safe distance for removing the lesion and the active infiltration zone at the edge of the lesion (12). A study from China has shown that there would be residual disease if the surgical margin was <10 mm, but the long-term survival rates could be nearly 100% by adding chemotherapy with Albendazole after surgery (13). Researchers have shown that the radical resection can be achieved at a distance of more than 10 mm (2). Therefore, in our study, the scope of 10 mm outside the lesion was defined as PLV.

HAE has a tendency to invade neighboring tissues like liver cancer (14). Similarly, it has been proposed that the area around the HAE lesion has aggregations of new blood vessels and immune cells, which serves as the basis for the alveolar hydatid to survive and infiltrate the surrounding area (15). However, it is impossible to give exact statement on the disease activity in AE without having a specimen on which to perform histological examination (16). How to obtain the bioactivity of HAE lesions under non-invasive conditions is worth considering. Imaging plays a pivotal role in the diagnosis and treatment of diseases; the new blood vessels can show enhancement after injection of the contrast agent (17). While CT and MRI can provide valuable information on disease distribution, morphological changes do not always accurately represent viable residual disease or can change only minimally even under response to treatment, thus hampering further treatment stratification (18). Functional imaging such as diffusion weighted imaging (DWI), perfusion-weighted imaging (PWI), contrast-enhanced ultrasound (CEUS), and PET/CT can detect the bioactivity of HAE to a certain extent; in particular, 18F-flurodeoxyglucose (18F-FDG) PET/CT was recommended by the WHO Informal Working Group to assess the activity of HAE (16–19). However, it is hard to popularize these functional imaging examinations in pastoral areas with high HAE incidence. In this study, we chose the most conventional CT portal phase images as the study sequence because it can clearly show the boundary of the lesion and the images are easy to obtain.

Radiomics has played a unique role in determining the heterogeneity of diseases, which has be used in diseases of various systems of the body (20, 21). In this study, GLV model, which was based on the VOI of the whole lesion, showed a good prediction effect. However, more and more studies have shown that the area around the lesion that appears macroscopically normal also has microscopic heterogeneity (22, 23). In our study, the PLV model based on the surrounding tissue of 10 mm had also achieved good results in predicting the bioactivity of HAE lesions. Delong test showed GPLV models, combined lesion and perilesion volume, had the best AUC (training, 0.868; validation, 0.777). This is consistent with a study on microvascular invasion in hepatocellular carcinoma (24). Not only in liver cancer but also in many other diseases in which studies have confirmed that the peritumoral model can be auxiliary to the intratumoral model so as to improve the application value of imaging radiomics in clinical work (25–28). This study is the first time to establish a PLV model in HAE. The PLV model performed well when applied alone; as expected, the prediction performance was greatly improved when it combined with GLV and clinical predictors. This also confirmed that there was heterogeneity in the microenvironment around the perilesional tissue of HAE.

Clinical features were analyzed in this study by univariate and multifactor logistic regression. Calcification type and fibrinogen had significant difference between the active group and inactive group in the training cohort. Moreover, this study combined the Radscore of the best radiomics model with clinical features to establish nomogram, which made the model visualization. Compared with the clinical model and GPLV model, nomogram showed the highest AUC (training, 0.914 vs. 0.868 vs. 0.754; validation, 0.833 vs. 0.777 vs. 0.719). DCA image showed that the nomogram had the best clinical application value.

Our study has several limitations. First, it is a single-center study with a relatively small sample size. Second, we only studied the PLV with 10 mm margin; it is necessary to build multiple models for further subdivision of the perilesion for precise diagnosis and treatment.

## 5 Conclusion

In conclusion, the nomogram model based on clinical and GPLV radiomic signatures shows the best performance in the prediction of the bioactivity of HAE. Radiomics including perilesional tissue can significantly improve the prediction efficacy of HAE bioactivity.

## Data availability statement

The datasets presented in this article are not readily available because we still have further research to do. Requests to access the datasets should be directed to SZ, 1830568214@qq.com.

## Ethics statement

The studies involving humans were approved by Ethics Committee of the First Affiliated Hospital of Xinjiang Medical University. The studies were conducted in accordance with the local legislation and institutional requirements. Written informed consent for participation was not required from the participants or the participants’ legal guardians/next of kin in accordance with the national legislation and institutional requirements.

## Author contributions

SZ: Resources, Methodology, Conceptualization, Writing – review & editing, Writing – original draft, Investigation, Data curation. JH: Validation, Supervision, Formal analysis, Writing – review & editing. WX: Investigation, Formal analysis, Data curation, Writing – review & editing. ZZ: Investigation, Writing – review & editing. MX: Supervision, Software, Methodology, Data curation, Conceptualization, Investigation, Writing – review & editing. SL: Writing – review & editing. CX: Writing – review & editing. TZ: Writing – review & editing, Investigation. WL: Writing – review & editing.
